# Comparison of in-hospital and out-of-hospital cardiac arrest of trauma patients in Qatar

**DOI:** 10.1186/s12245-022-00454-0

**Published:** 2022-09-16

**Authors:** Furqan B. Irfan, Rafael I. G. D. J. Consunji, Ruben Peralta, Ayman El-Menyar, Landric B. Dsouza, Jassim M. Al-Suwaidi, Rajvir Singh, Maaret Castrén, Therese Djärv, Guillaume Alinier

**Affiliations:** 1 Department of Neurology and Ophthalmology, College of Osteopathic Medicine, Institute of Global Health, Michigan State University, West Fee Hall, 909 Wilson Road, East Lansing, MI 48824 USA; 2 Hamad Trauma Center at Hamad General Hospital, Hamad Medical Corporation, Doha, Qatar; 3 Department of Surgery, School of Medicine, Universidad Nacional Pedro Henriquez Urena, Santo Domingo, Dominican Republic; 4 Weill Cornell Medicine – Qatar, Education City, Doha, Qatar; 5 Department of Emergency Medicine, Hamad General Hospital, Hamad Medical Corporation, Doha, Qatar; 6 Heart Hospital, Hamad Medical Corporation, Doha, Qatar; 7 Department of Emergency Medicine and Services, University of Helsinki, Helsinki, Finland; 8 Karolinska Institutet, Stockholm, Sweden; 9 Hamad Medical Corporation - Ambulance Service, Al Rayyan Road, Doha, Qatar; 10 School of Health and Social Work, University of Hertfordshire, College Lane, HERTS, Hatfield, AL10 9AB UK; 11 Faculty of Health and Life Sciences, Coach Lane Campus, Northumbria University, Newcastle, Newcastle upon Tyne, NE7 7TR UK

**Keywords:** In-hospital cardiac arrest of trauma patient, Qatar, Patient outcome, Mortality, Survival, Trauma

## Abstract

**Background:**

Cardiac arrests in admitted hospital patients with trauma have not been described in the literature. We defined “in-hospital cardiac arrest of a trauma” (IHCAT) patient as “cessation of circulatory activity in a trauma patient confirmed by the absence of signs of circulation or abnormal cardiac arrest rhythm inside a hospital setting, which was not cardiac re-arrest.” This study aimed to compare epidemiology, clinical presentation, and outcomes between in- and out-of-hospital arrest resuscitations in trauma patients in Qatar. It was conducted as a retrospective cohort study including IHCAT and out-of-hospital trauma cardiac arrest (OHTCA) patients from January 2010 to December 2015 utilizing data from the national trauma registry, the out-of-hospital cardiac arrest registry, and the national ambulance service database.

**Results:**

There were 716 traumatic cardiac arrest patients in Qatar from 2010 to 2015. A total of 410 OHTCA and 199 IHCAT patients were included for analysis. The mean annual crude incidence of IHCAT was 2.0 per 100,000 population compared to 4.0 per 100,000 population for OHTCA. The univariate comparative analysis between IHCAT and OHTCA patients showed a significant difference between ethnicities (*p*=0.04). With the exception of head injury, IHCAT had a significantly higher proportion of localization of injuries to anatomical regions compared to OHTCA; spinal injury (OR 3.5, 95% CI 1.5–8.3, *p*<0.004); chest injury (OR 2.62, 95% CI 1.62–4.19, *p*<0.00), and abdominal injury (OR 2.0, 95% CI 1.0–3.8, *p*<0.037). IHCAT patients had significantly higher hypovolemia (OR 1.66, 95% CI 1.18–2.35, *p*=0.004), higher mean Glasgow Coma Scale (GCS) score (OR 1.4, 95% CI 1.3–1.6, *p*<0.00), and a greater proportion of initial shockable rhythm (OR 3.51, 95% CI 1.6–7.7, *p*=0.002) and cardiac re-arrest (OR 6.0, 95% CI 3.3–10.8, *p*=<0.00) compared to OHTCA patients. Survival to hospital discharge was greater for IHCAT patients compared to OHTCA patients (OR 6.3, 95% CI 1.3–31.2, *p*=0.005).

Multivariable analysis for comparison after adjustment for age and gender showed that IHCAT was associated with higher odds of spinal injury, abdominal injury, higher pre-hospital GCS, higher occurrence of cardiac re-arrest, and better survival than for OHTCA patients.

IHCAT patients had a greater proportion of anatomically localized injuries indicating solitary injuries compared to greater polytrauma in OHTCA. In contrast, OHTCA patients had a higher proportion of diffuse blunt non-localizable polytrauma injuries that were severe enough to cause immediate or earlier onset of cardiac arrest.

**Conclusion:**

In traumatic cardiac arrest patients, IHCAT was less common than OHTCA and might be related to a greater proportion of solitary localized anatomical blunt injuries (head/abdomen/chest/spine). In contrast, OHTCA patients were associated with diffuse blunt non-localizable polytrauma injuries with increased severity leading to immediate cardiac arrest. IHCAT was associated with a higher mean GCS score and a higher rate of initial shockable rhythm and cardiac re-arrest, and improved survival rates.

## Introduction

Trauma patients suffering cardiac arrest in-hospital have not been previously described. The epidemiology and outcomes of trauma patients following in-hospital cardiac arrest may differ significantly from out-of-hospital traumatic cardiac arrest (OHTCA). To our knowledge, this has not been specifically described elsewhere and this may represent an opportunity to provide evidence for clinical algorithms, quality improvement, and prognostication of patient outcomes. We defined in-hospital cardiac arrest of a trauma (IHCAT) patient as “cessation of circulatory activity in a trauma patient confirmed by the absence of signs of circulation or abnormal cardiac arrest rhythm in a hospital setting, following a traumatic injury, and was not cardiac re-arrest.”

Pre-hospital or OHTCA can be defined, as cessation of circulatory activity in a trauma patient that is confirmed by the absence of signs of circulation and that occurs outside of a hospital setting [1–3]. Recent studies have reported the incidence of out-of-hospital traumatic cardiac arrest to be 6.0 per 100,000 and as high as 45.7 per 100,000 with survival rates ranging from 1.6 to 32% [4–10]. The International Liaison Committee on Resuscitation (ILCOR), in 2010, issued guidelines on emergency management of traumatic cardiac arrest [11, 12]. Recent European Resuscitation Council Guidelines 2021, have improved on the 2015 guidelines, with traumatic cardiac arrest/peri-arrest algorithm: sonography for diagnostic evaluation; simultaneous treatment of reversible causes (uncontrolled hemorrhage, tension pneumothorax, asphyxia, pericardial tamponade) is prioritized over chest compressions; external hemorrhage control; resuscitative thoracotomy and Resuscitative Endovascular Balloon Occlusion of the Aorta (REBOA) for uncontrollable infradiaphragmatic hemorrhage; bilateral thoracostomies leading to clamshell thoracotomy for chest decompression; pelvic splint; and blood products/massive hemorrhagic protocol [13].

We have previously described and determined predictors of survival in a nationwide population-based study on OHTCA, in Qatar [14]. This study aims to describe epidemiology, clinical presentation, and outcomes of IHCAT in comparison to OHTCA. Given the hypothesis, this study had the following objectives: firstly, to describe epidemiology, peri-cardiac arrest and injury characteristics, and outcomes of IHCAT; and secondly to compare IHCAT with OHTCA.

## Methods

### Study design and population

This was a retrospective cohort study that included IHCAT and OHTCA patients from January 2010 to December 2015, and utilized prospectively collected data from the national trauma registry, the out-of-hospital cardiac arrest registry, and national Hamad Medical Corporation Ambulance Service (HMCAS) database, in Qatar [15]. The national trauma registry and out-of-hospital cardiac arrest registry have been described in detail previously [16]. HMCAS is the sole provider of Emergency Medical Services (EMS) in Qatar, is well equipped, and maintains a database of all patients managed in the pre-hospital setting [17, 18]. The results of OHTCA patients have been reported in a previous nationwide population-based study and have been used for comparison with IHCAT patients in this study [14]. IHCAT and OHTCA patients were linked by their medical record number which was consistent across the three databases. Patient medical record files and electronic medical records were reviewed to collect data on all patients included in the study. Ethics approval and waiver of informed consent were obtained from the Institutional Review Board of Hamad Medical Corporation (JIRB# 13-00071 and JIRB# 14384/14). The study was performed in accordance with the Declaration of Helsinki.

### Measures

#### Exposure

The inclusion criteria included adult (greater than 18 years) IHCAT patients with evidence of absence of signs of circulation or abnormal cardiac arrest rhythm, following a traumatic injury, and having received cardiopulmonary resuscitation (CPR) in the emergency department (ED) or hospital. There was no time limit to the interval between the initial trauma and the in-hospital cardiac arrest.

OHTCA patients were the comparative group and included adult trauma patients with pre-hospital cardiac arrest determined by EMS and having received pre-hospital CPR.

Exclusion criteria for both groups included trauma patients with clear signs of death and injuries due to drowning, electrocution, or burns.

### Covariates and outcome

Variables considered included demographic characteristics, trauma clinical presentation, pre-hospital interventions, in-hospital cardiac arrest features, and ED/trauma room and in-hospital management. Demographic characteristics included age, gender, and ethnicity. Ethnicity was classified as Middle Eastern, Caucasians, South Asian, Far Eastern, and African. Trauma and injury features included mechanism of injury (motor vehicle collision, falls, pedestrian, and others including assaults and gunshot wounds), type of injury (blunt vs. penetrating), anatomical region of injury (chest, abdomen, head, and spine), and pre-hospital Glasgow Coma Scale (GCS). Etiology of traumatic cardiac arrest was considered from the treating physician’s notes and included hypoxia, hypovolemia, and severe head injury. Peri-cardiac arrest features included initial shockable rhythm and cardiac re-arrest.

Pre-hospital bystander interventions that were particular to OHTCA patients like bystander witnessed cardiac arrest, bystander CPR, and bystander defibrillation were not included. EMS pre-hospital management (airway, adrenaline administration, etc.), and ED and in-hospital management (thoracotomy, blood transfusion, etc.) were also not considered because these were location specific and time-dependent for OHTCA and IHCAT patients. The main outcome assessed was an association of covariate variables with OHTCA and IHCAT.

### Statistical analysis

Given the rapid development of the country and its population, the mid-year population over each year of the 6-year study period was used to calculate the mean incidence rate for IHCAT [14]. Univariate analysis included frequencies and percentages for categorical variables and means with standard deviations or medians with interquartile ranges for continuous variables. Bivariate association of IHCAT and OHTCA with covariates and outcomes were determined using chi-square for categorical variables and *t*-test or Mann-Whitney test for continuous variables. Multivariable step-wise forward logistic regression models were created to control for confounding factors. A cut-off p-value of less than 0.1 in univariate analysis was used to include variables in the multivariable models. P-value 0.05 (two-tailed) was used for statistical significance. Area under the receiver operator characteristic (ROC) curve was used to assess the model. Hosmer–Lemeshow goodness-of-fit test was used to determine the correct specification of the model. Statistical analysis was performed using SPSS (IBM SPSS Statistics version 23.0).

## Results

**Fig. 1:**
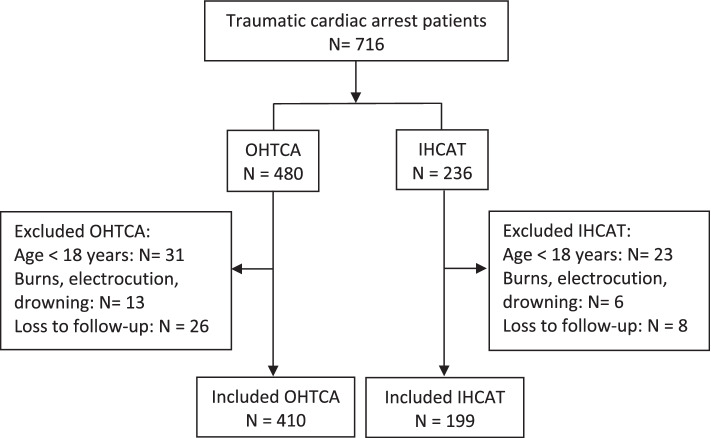
Traumatic cardiac arrest (OHTCA and IHCAT) patients

There were a total of 716 traumatic cardiac arrests in Qatar from 2010 to 2015. Of these, 480 had OHTCA while 236 patients had IHCAT. After exclusion, a total of 410 OHTCA patients and 199 IHCAT patients were included for analysis (Fig. 1).

The mean annual crude incidence rate of IHCAT was 2.0 per 100,000 population. There was a predominance of male (*n*= 180, 90.4%) IHCAT patients with a median age of 33.5 (IQR 25–48.3). South Asians (*n*=92, 46.2%) had the highest number of IHCAT followed by Middle Easterners (*n*=60, 30.2%) and other ethnicities. Most of the IHCAT were blunt injuries (*n*=191, 96.0%) that occurred on streets and roads (*n*=146, 73.4%). Road traffic injuries were the leading cause of IHCAT including motor vehicle collisions (*n*=77, 38.7%), pedestrians (*n*=55, 27.6%), and cyclists and motorcyclists (*n*=5, 2.5%). The major anatomical injury regions for IHCAT patients included the head (*n*=120, 60.3%); abdomen (*n*=70, 35.2%); chest (*n*=44, 22.1%); and spine (*n*=40, 20.1%). Etiology of IHCAT assessed included hypovolemia (*n*=91, 45.7%) and hypoxia (*n*=84, 42.4%). The mean pre-hospital GCS was 6.56 ± 4.68. Initial shockable rhythm (ventricular fibrillation and ventricular tachycardia) was present in 15 (7.5%) IHCAT patients. Cardiac re-arrest occurred in 103 (51.8%) IHCAT patients. Survival to hospital discharge rate was 7.5% (*n*=15) for IHCAT patients (Table 1).

**Table 1:** Comparison of OHTCA and IHCAT patients

Variable	OHTCA ***N***=410	IHCAT ***N***=199	Unadjusted odds ratio (95% CI) ***p***-value	Adjusted odds ratio (95% CI) ***p***-value
**Demographics**				
**Age** (median, IQR) (Mann-Whitney *U t*-test)	33.0 (27–46)	33.5 (25–48.3)	1.0 (0.99–1.02) *p* = 0.83	**-**
Missing	118 (28.8%)	29 (14.6%)		
**Gender**				
Male	377 (91.9%)	180 (90.4%)	Reference	
Female	29 (7.1%)	17 (8.5%)	0.82 (0.44–1.52) *p* = 0.52	
Missing	4 (1.0%)	2 (1.0%)		
**Location**			Overall *p* = 0.47	-
Road	303 (74.0%)	146 (73.4%)	Reference	
Home	21 (5.1%)	15 (7.5%)	1.48 (0.74–2.96) *p* = 0.26	
Workplace	42 (10.2%)	22 (11.1%)	1.09 (0.62–1.89) *p* = 0.77	
Public place	40 (9.8%)	14 (7.1%)	0.73 (0.38–1.38) *p* = 0.33	
Missing	4 (1.0%)	2 (1.0%)		
**Ethnicity**			**Overall** ***p*** **= 0.04**	-
Middle Eastern	103 (25.1%)	60 (30.2%)	Reference	
Caucasian	10 (2.4%)	2 (1.0%)	0.34 (0.07–1.62) *p* = 0.18	
South Asian	154 (37.6%)	92 (46.2%)	1.03 (0.68–1.55) *p* = 0.90	
Far Eastern	12 (3.0%)	17 (8.5%)	**2.43 (1.09–5.44)** ***p*** **= 0.03**	
African	15 (3.7%)	4 (2.0%)	0.46 (0.15–1.44) *p* = 0.18	
Missing	116 (28.3%)	24 (12.1%)		
**Injury characteristics**				
**Mechanism of injury**			Overall *p* = 0.33	-
Motor vehicle collision	172 (42.0%)	77 (38.7%)	Reference	
Falls	57 (13.9%)	41 (20.6%)	**1.61 (1.0–2.60)** ***p*** **= 0.05**	
Pedestrian	119 (29.0%)	55 (27.6%)	1.03 (0.68–1.57) *p* = 0.88	
Vulnerable road users	14 (3.4%)	5 (2.5%)	0.80 (0.28–2.30) *p* = 0.68	
Others	48 (11.7%)	21 (10.6%)	0.98 (0.55–1.74) *p* = 0.94	
**Type of injury**				
Blunt	383 (93.4%)	191 (96.0%)	Reference	
Penetrating	23 (5.6%)	7 (3.5%)	0.61 (0.26–1.45) *p* = 0.26	
Missing	4 (1.0%)	1 (0.5%)		
**Head injury**	271 (66.1%)	120 (60.3%)	0.77 (0.54–1.1) *p* = 0.15	-
Missing	1 (0.2%)	0		
**Spinal injury**	28 (6.8%)	40 (20.1%)	**3.43 (2.04–5.76)** ***p*** **<0.00**	**3.5 (1.5–8.3)** ***p*** **= 0.004**
**Chest injury**	40 (9.8%)	44 (22.1%)	**2.62 (1.64–4.19)** ***p*** **<0.00**	-
**Abdominal injury**	67 (16.3%)	70 (35.2%)	**2.78 (1.88–4.11)** ***p*** **<0.00**	**2.0 (1.0–3.8)** ***p*** **= 0.037**
**Hypovolemia**	138 (33.7%)	91 (45.7%)	**1.66 (1.18–2.35)** ***p*** **= 0.004**	-
**Hypoxia**	186 (45.4%)	84 (42.4%)	0.88 (0.63–1.24) *p* = 0.46	-
**Pre-hospital GCS (Mean ± SD)**	3.44 ± 1.9	6.56 ± 4.68	**1.34 (1.25–1.43)** ***p*** **<0.00**	**1.4 (1.3–1.6)** ***p*** **<0.00**
Missing	19 (4.6%)	0		
**Cardiac arrest features**				
**Initial shockable rhythm**	12 (2.9%)	15 (7.5%)	**3.51 (1.6–7.7)** ***p*** **= 0.002**	-
Missing	61 (14.9%)	64 (32.2%)		
**Cardiac re-arrest**	97 (23.7%)	103 (51.8%)	**3.36 (2.3–4.8)** ***p*** **<0.00**	**5.97 (3.3–10.8)** ***p*** **<0.00**
Missing	9 (2.2%)	0		
**Outcome**				
**Survival**	10 (2.4%)	15 (7.5%)	**3.26 (1.4–7.4)** ***p*** **= 0.005**	**6.3 (1.3– 31.2)** ***p*** **= 0.025**

The univariate analysis of the comparison between IHCAT and OHTCA patients showed an overall significant difference between ethnicities (*p* = 0.04); a higher proportion of IHCAT patients were of Far Eastern ethnicity (8.5%) compared to OHTCA patients (3.0%) (*p* = 0.03). The mechanism of injury in IHCAT had a higher proportion of falls (20.6%) compared to OHTCA (13.9%) (*p* = 0.05). With the exception of head injury, IHCAT patients had a significantly higher proportion of injuries localized to specific anatomical regions compared to OHTCA; spinal injury (p <0.00); chest injury (*p* <0.00), and abdominal injury (*p* <0.00). A higher proportion of IHCAT (45.7%) compared to OHTCA (33.7%) patients (*p* = 0.004) had hypovolemia. IHCAT patients had a significantly higher mean GCS score (6.56) compared to OHTCA patients (3.44) (*p* <0.00). IHCAT patients had a greater proportion of initial shockable rhythm (*p* = 0.002) and cardiac re-arrest (*p* = <0.00) compared to OHTCA patients. Survival to hospital discharge was greater for IHCAT (7.5%) compared to OHTCA (2.4%) patients (*p* = 0.005) (Table 1).

Multivariable analysis after adjustment for age and gender showed that IHCAT was associated with greater odds of spinal injury (OR 3.5, 95% CI 1.5–8.3, *p* = 0.004), chest injury (OR 2.62, 95% CI 1.64–4.19, *p*<0.00), abdominal injury (OR 2.0, 95% CI 1.0–3.8, *p* = 0.037), hypovolemia (OR 1.66, 95% CI 1.18–2.35, *p*<0.00), cardiac re-arrest (OR 6.0, 95% CI 3.3–10.8, *p* <0.00), higher pre-hospital GCS (OR 1.4, 95% CI 1.4–1.6, *p*<0.00), and better survival (OR 6.3, 95% CI 1.3–31.2, *p* = 0.025) (Table 1).

## Discussion

To the best of our knowledge, this is the first study to describe the characteristics of IHCAT and comparing OHTCA and IHCAT. Previous studies of OHTCA or pre-hospital traumatic cardiac arrest have included trauma patients with evidence of pre-hospital cardiac arrest or CPR [19]. However, some traumatic cardiac arrest studies have combined patients, defined as CPR either in the pre-hospital phase and/or during trauma room treatment [9, 20]. Few studies have reported on cardiac arrest in trauma patients in ED only [9, 21]. None of the studies, however, has designated IHCAT as a separate study population with different pathophysiology of delayed cardiac arrest occurring in-hospital in a trauma patient.

The majority of the traumatic cardiac arrest studies have included patients either from a cardiac arrest registry or a trauma registry [19, 22]. We defined traumatic cardiac arrest to include IHCAT and OHTCA and demonstrated that IHCAT is a distinct type of traumatic cardiac arrest that is different from OHTCA in terms of epidemiology, etiology, pathophysiology, and outcomes.

IHCAT patients have delayed cardiac arrest in the ED and in-hospital phase compared to OHTCA patients who suffer cardiac arrest in the pre-hospital phase. The postulated pathophysiology of delayed cardiac arrest in IHCAT patients is varied and further studies need to be undertaken to determine the causes of their delayed cardiac arrest. The trauma in IHCAT patients always precedes the cardiac arrest in contrast to OHCTA which may include some cases where cardiac arrest precedes secondary trauma. It is unclear how many of the OHTCA cases might be primarily cardiac in origin (i.e., a cardiac event that precedes or causes the traumatic event).

In contrast to previous literature that bemoans the “dismal” survival of trauma patients who suffer from a cardiac arrest, our results show these patients are actually 2 distinct populations with significantly different survival rates, more so after adjusting for confounders [23]. For trauma systems, we have reiterated the importance of getting the patient to the “right care in the right time,” as well as ‘scoop and run’ rather than ‘stay and play.’ A patient who arrests in a trauma center has 6 times higher odds of survival when compared to one that arrests in the pre-hospital phase. For clinical prognostication, we have shown that a trauma patient who arrests, in-hospital, does not have as dismal prognosis as reported in previous literature that did not differentiate this entity from OHTCA. For future reports on trauma patient survival from arrest, we recommend that the practice of ‘lumping’ together all trauma patients be stopped and all the proper classification of such patients be made.

The mean annual crude incidence of IHCAT was 2.0 per 100,000 population compared to 4.0 per 100,000 population for OHTCA, in Qatar. The incidence of cardiac arrest in trauma patients is significant such that it should be recognized as a separate sub-type of cardiac arrest and not simply as an “arrest in special situations.” Since there are no studies of IHCAT, we could not compare the incidence rates with other populations. However, the mean annual crude incidence of OHTCA in Melbourne, for example, was 6.0 per 100,000 [8]. Extrapolating our results, the incidence of IHCAT would be half of OHTCA; giving an incidence of 3.0 per 100,000 for IHCAT in Melbourne.

There were no significant differences noted in the demographic nor injury characteristics of the OHTCA and IHCAT patients. Those that seemed initially significant, i.e. Far Eastern ethnicity, did not persist after multi-variate adjustment. This is different from another study of all out-of-hospital cardiac arrest (OHCA) in Qatar concentrating only on Middle Eastern Arabs and North Africans, and which found that the latter had better chances of survival as they were generally younger with decreased risk factors, more favorable cardiac rhythms, shorter EMS response times, received more Advanced Cardiac Life Support (ACLS) interventions and had longer scene times [24]. The consistent predominance of males, South Asian, and Middle Eastern populations in this study population is simply reflective of the demographic breakdown of the national population of Qatar [25].

The interval between initial trauma and IHCAT is expected to follow the pattern of trauma death described by Sauaia et al., in the USA, and Abdelrahman et al., in Qatar [26, 27]. This work takes the analysis further by separating pre- and in-hospital arrests in trauma patients, therefore providing the evidence that more than two-thirds (67%) of all traumatic arrests, in Qatar during the study period, occur in the pre-hospital setting. This has major implications for healthcare policy prioritization and future research. More resources must be earmarked for quality improvement in pre-hospital care and the primary prevention of trauma through proven programs that will prevent the leading causes of OHTCA [28–30].

Road traffic injuries were the leading mechanism of injury of IHCAT and OHTCA patients. However, IHCAT patients had higher odds of injury due to ‘falls,’ compared to OHTCA patients, in univariate analysis. Greater than ninety percent of OHTCA and IHCAT patients had blunt injuries. The high percentage of blunt injuries was similar to OHTCA injury pattern with very few assaults and gunshot penetrating injuries. (9) This may be unique to Qatar, where trauma registry data over the years has shown a less than 5% of injuries due to a penetrating mechanism (ACS TQIP Benchmark report: Fall 2020. American College of Surgeons, Committee on Trauma, Trauma Quality Improvement Program, 2021) [31, 32].

Patients with IHCAT were associated with delayed cardiac arrest in ED and in-hospital.

IHCAT patients had a greater proportion of anatomically localized injuries indicating solitary injuries compared to greater polytrauma in OHTCA. In contrast, OHTCA patients had a higher proportion of diffuse blunt non-localizable polytrauma injuries that were severe enough to cause immediate or earlier onset of cardiac arrest.

The difference in anatomical injury pattern was statistically significant for a spinal injury and abdominal injury that persisted after multivariable analysis. Abdominal and spinal injury patterns may indicate different pathophysiological pathways, for example, involving the thoracoabdominal autonomic nervous system causing bradycardia leading to cardiac arrest [33]. These two isolated anatomical injury patterns may also be surrogate indicators for less immediately fatal mechanisms of injury in IHCAT patients leading to delayed onset of cardiac arrest in the hospital.

Despite a comparable proportion of head injuries, the mean GCS score for IHCAT was nearly twice that for OHTCA patients. The difference in mean GCS score was statistically significant even after adjustment and underlines the finding that OHTCA patients had more severe traumatic brain injury (TBI) and were more likely to be recipients of greater energy transfer during the initial traumatic event. Again, this has implications for efforts to prevent this energy transfer for the most common mechanisms of injury (i.e. seatbelts and helmets for motor vehicle collisions [MVC] or helmets and harnesses for falls) and what treatment or interventions they may benefit from in the pre-hospital setting and once in the ED or Trauma Center [34, 35].

Hypoxia secondary to airway and respiratory compromise in OHTCA and IHCAT patients were comparable. Hypovolemia had a statistically significantly higher proportion in IHCAT patients compared to OHTCA patients that did not persist after adjustment. Hypovolemia, as a result of massive hemorrhage, is either massive and lethal at the scene (i.e., aortic transection) or is initially survivable but aggravated by the time it is left unidentified as the fluids infused and the coagulopathy of trauma ensues. It is the latter that would comprise the population of IHCAT patients with hemorrhagic shock.

Overall, the proportions of OHTCA and IHCAT patients with an initial shockable rhythm was low, 2.9% and 7.5%, respectively. While these proportions were much lower than what we noted for presumed cardiac origin OHCA, 19.7%, an initial shockable rhythm still predicted a more favorable outcome for both.

IHCAT was associated with a greater proportion of cardiac re-arrest after adjustment. Cardiac re-arrest has been reported to be associated with decreased survival in OHCA patients [36]. In contrast, this study determined that cardiac re-arrest was associated with IHCAT patients who had greater survival compared to OHTCA patients. Further studies are required to explore re-arrest in traumatic cardiac arrest (TCA), OHTCA, and IHCAT.

After multivariate adjustment, IHCAT patients had higher survival rates compared to OHTCA. The Donabedian model for healthcare quality measures in this setting emphasizes that even in the presence of structural measures, including EMS personnel that are not only BLS/ACLS/ATLS certified but are even augmented by critical care paramedics, there is a survival benefit to expediting transport to a trauma center [15, 37]. This highlights the need for prevention and process measures, the implementation of proven interventions that will prevent the crashes and falls from occurring in the first place or reduce the force/s that are imparted on the victims, i.e., speed limits, high-visibility law enforcement, and enhanced seat belt laws for MVCs and tighter enforcement of occupational safety regulations on safety harnesses and helmets for falls [30, 34, 35].

## Limitations

We noted a higher proportion of initial shockable rhythm in IHCAT patients. One reason is that all initial rhythms in IHCAT patients were recorded since the cardiac arrests occurred in ED or in-hospital, while for OHTCA patients what may have been an initial shockable rhythm with conversion became a non-shockable rhythm before EMS had the time to arrive, record it, and initiate the appropriate treatment to convert it.

Only association could be inferred and not causality, since it was an observational retrospective cohort study. Identification bias was minimized by identifying traumatic cardiac arrest, both IHCAT and OHTCA patients from multiple sources including the national ambulance service, cardiac arrest registry, and trauma registry. Additionally, a few traumatic cardiac arrest patients that did not utilize the EMS and died pre-hospital may have been missed. Etiology of traumatic cardiac arrest could only be ascertained clinically in the absence of an autopsy as is the case with the majority of cardiac arrest studies. Trauma severity, by using Injury Severity Score, could not be determined and would have been especially important in this study, since IHCAT patients had less severe injuries, in view of the association with higher mean GCS score, initial shockable rhythm, and survival.

## Conclusions

Trauma patients who suffer a cardiac arrest do not necessarily have a worse prognosis and must be classified into two distinct categories; OHTCA and IHCAT. IHCAT was less common than OHTCA, associated with delayed cardiac arrest and with injuries anatomically localized to the head, abdomen, chest, and spine. IHCAT was associated with a higher mean GCS score and initial shockable rhythm and cardiac re-arrest and improved survival rates. Survival rates of IHCAT were comparable to medical cardiac arrest and should be aggressively managed.

## Data Availability

All anonymized data are available from FBI subject to permission from Hamad Medical Corporation.

## Abbreviations

ACLS: Advanced Cardiac Life Support
ATLS: Advanced Trauma Life Support
BLS: Basic life support
CPR: Cardiopulmonary resuscitation
ED: Emergency department
EMS: Emergency Medical Services
GCS: Glasgow Coma Scale
HMCAS: Hamad Medical Corporation Ambulance Service
IHCAT: In-hospital cardiac arrest of a trauma
ILCOR: International Liaison Committee on Resuscitation
IQR: Interquartile range
MVC: Motor vehicle collision
OHTCA: Out-of-hospital traumatic cardiac arrest
OR: Odds ratio
ROC: Receiver operator characteristic
SD: Standard deviation
TBI: Traumatic brain injury
TCA: Traumatic cardiac arrest
